# Supplementary material for: Complexome profiling of the Chlamydomonas psb28 mutant reveals TEF5 as an early PSII assembly factor
Source: Plant Cell. 2025 Mar 17;37(6):koaf055. doi: 10.1093/plcell/koaf055 (PMC12164590; doi:10.1093/plcell/koaf055)
Supplement: koaf055_Supplementary_Data [file koaf055_supplementary_data.zip › psb28_interactive.zip!/plots/intensity/qualityPlots/peptides/_Cre01.g007700.t1.1.html]

### Cre01.g007700.t1.1

Trivial name:   
SeqCoverage [median]: 7.90  
Mass: 62.26 kDa  
  
 

| Sequence | MissedCleavage | Mass [Da] | wtIntensity | muIntensity | Score |
| --- | --- | --- | --- | --- | --- |
| AGAGSMIELMK | 0 | 1106.55 | 0.00 | 317.14 | 56.73 |
| GLTFDSGGYNLK | 0 | 1270.62 | 0.00 | 1519.96 | 89.25 |
| TVEVNNTDAEGR | 0 | 1303.60 | 397.97 | 2014.38 | 132.91 |
|  |  |  | 398.0 | 3851.5 |  |
